# Cold Agglutinin Disease: A Rare Paraneoplastic Manifestation of a Thyroid Malignancy

**DOI:** 10.7759/cureus.67924

**Published:** 2024-08-27

**Authors:** Abhishek Pandey, Disha Arora, Arun Singh, Lalit Prashant Meena

**Affiliations:** 1 Medicine, Institute of Medical Sciences, Banaras Hindu University, Varanasi, IND; 2 Department of Endocrinology, Sapthagiri Institute of Medical Sciences & Research Centre, Bengaluru, IND; 3 Nephrology, Atal Bihari Vajpayee Institute of Medical Sciences and Dr. Ram Manohar Lohia Hospital, New Delhi, IND

**Keywords:** thyroid nodule malignancy, hurthle cell carcinoma, oncocytic thyroid carcinoma, paraneoplastic manifestation, cold agglutinin disease

## Abstract

A paraneoplastic syndrome is the presence of signs and symptoms due to cancer, but it is not a consequence of the mass effect of a tumour. It typically occurs in middle-aged to older patients with solid tumors (lung, breast, and ovaries), and hematological malignancies (leukemia and lymphoma).

Autoimmune hemolytic anaemia is also a well-defined paraneoplastic phenomenon in lymphoproliferative disorders and rare solid tumour malignancies such as renal cell carcinoma, ovarian dermoid cysts, thymus cell cancer, Kaposi sarcoma, and cancers of the breast, pancreas, thyroid, and prostate. Most of the time, it is warm and is rarely cold type. We present a case of cold-type autoimmune hemolytic anaemia, presented as paraneoplastic manifestations of a thyroid malignancy.

## Introduction

Cold agglutinin disease (CAD) is a type of autoimmune hemolytic anaemia (AIHA) in which clinical symptoms are caused by a cold-sensitive autoantibody at a temperature of 3-4°C. This autoantibody is usually an IgM-type antibody directed against the I antigen of the RBC surface. Hemolysis is extravascular and complement-mediated. CAD can occur primarily (idiopathic) or secondary to an infection or malignancy, usually a lymphoproliferative disorder, and rarely, a solid tumour malignancy. We report a case of CAD in a patient of mixed-histology thyroid malignancy (follicular carcinoma and follicular variant of papillary carcinoma), which can be a paraneoplastic manifestation of an underlying tumour.

## Case presentation

A 50-year-old female presented with fever, yellowish discolouration of the sclera, and dark urine for 14 days. Fever was intermittent, with a maximum recorded of 40°C. Jaundice was associated with pain in the abdomen but not associated with hematemesis or the passage of clay-coloured stools and pruritus. She developed progressive breathlessness on exertion along with generalised body weakness and easy fatiguability subsequently. On examination, the patient had pallor, icterus, and hepatosplenomegaly. Incidentally, she had a firm, nodular swelling on the right side of the midline of 4 x 4 cm with an upward extension 2 cm below the superior angle of the thyroid and a downward extension 2 cm above the suprasternal notch. It was moving with deglutination but not with the protrusion of the tongue, suggesting thyroid swelling. An important finding was that her blood sample was clotted as soon as it was withdrawn.

Investigations

Routine investigation showed haemoglobin was 6.1 g/dl, mean corpuscular volume (MCV) was 107 fl, mean corpuscular haemoglobin (MCH) was 92, mean corpuscular haemoglobin concentration (MCHC) was 976 gm/dl, RBC count was 0.06 million per mm3 with a red blood cell distribution width (RDW) of 25%, total leucocyte count was 13,000 per microliter with 38% neutrophils, 55% lymphocytes, and the platelet count was 3,80,000 platelets/µL. Transaminases SGOT and SGPT were elevated at 410 and 560 U/L, respectively. Total bilirubin was 5.1 mg/dL, with a predominant indirect fraction of 4.1 mg/dL. The lactate dehydrogenase level was 980 U/L, alkaline phosphatase was 398 U/L, and gamma-glutamyl transferase (GGT) was 95 U/L. These elevated levels of transaminases, bilirubin, lactate dehydrogenase, alkaline phosphatase, and gamma-glutamyl transferase indicated significant liver dysfunction. The predominant indirect bilirubin fraction suggested the possibility of hemolysis. Despite the liver abnormalities, the normal blood urea, serum creatinine, and electrolyte levels indicated that kidney function appeared to be unaffected. The tests for malaria and leptospirosis were negative.

**Figure 1:**
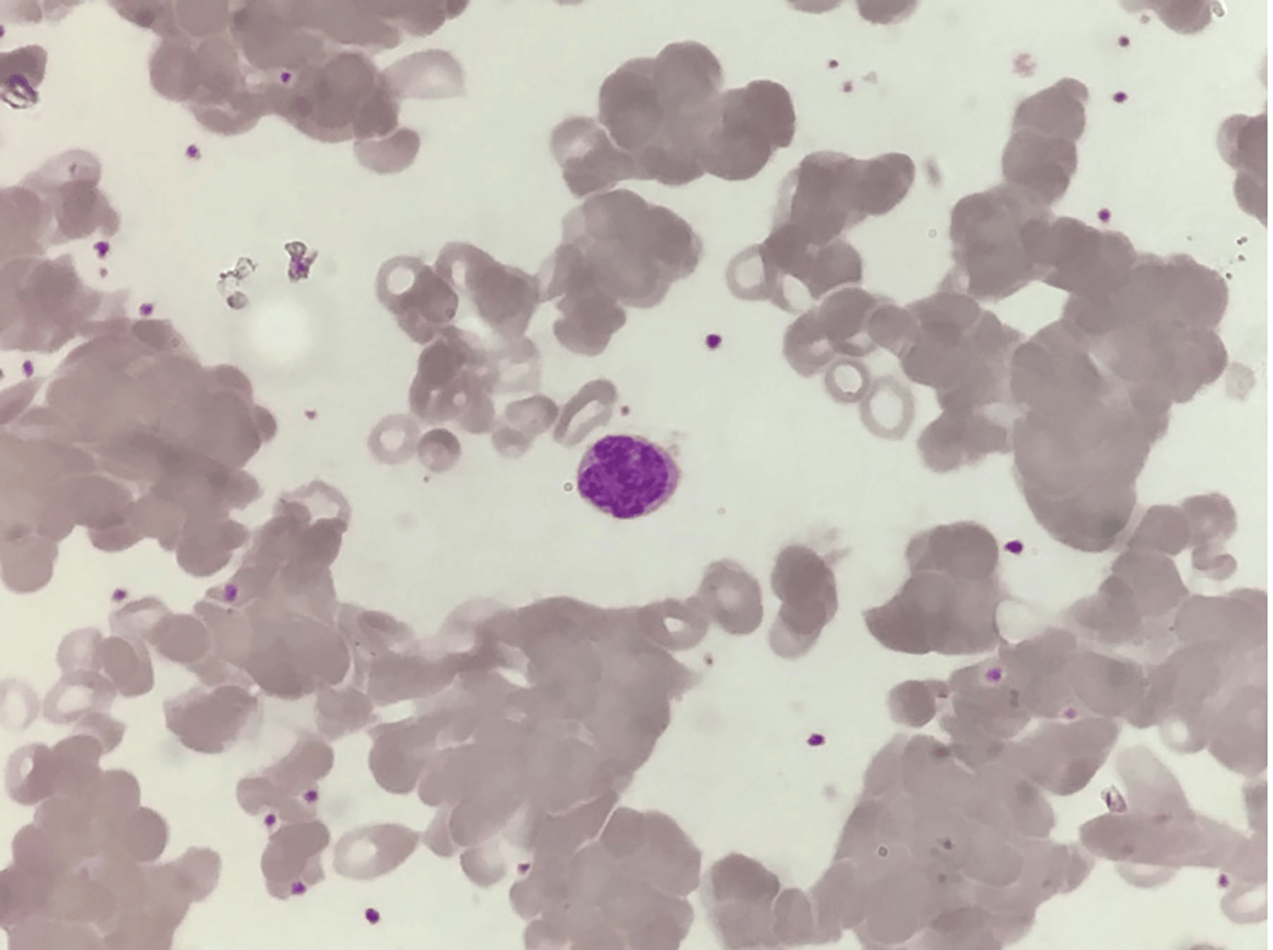
Peripheral blood smear at 100x magnification on Leishman's stain showing agglutinated masses of RBCs of variable size. Both the WBC and platelet counts were normal.

**Figure 2:**
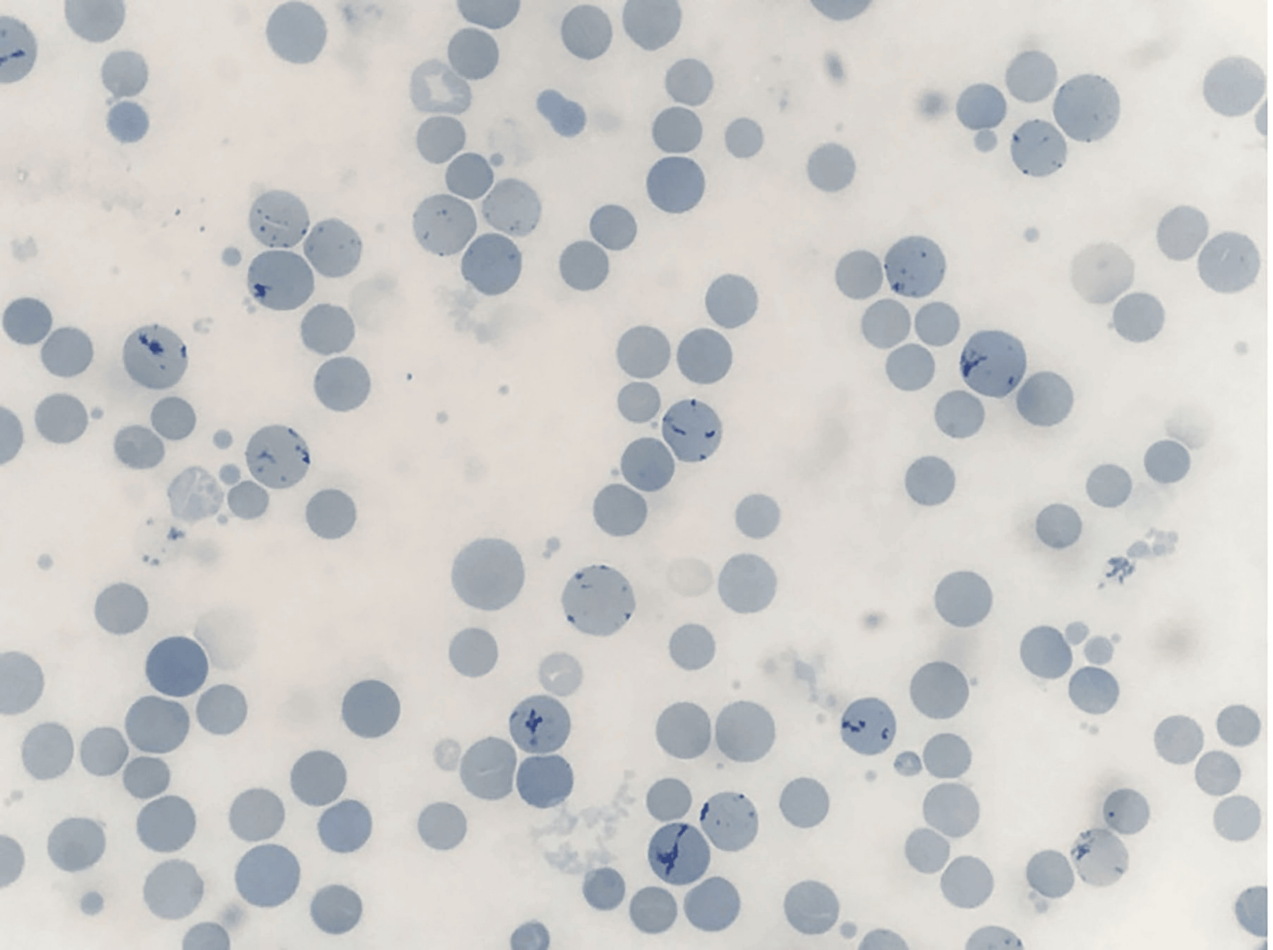
Peripheral blood smear at 100x magnification on a supravital stain showing reticulocytosis.

She was euthyroid, and anti-thyroid peroxidase (anti-TPO) was negative. Both acute and chronic viral markers were negative. Infectious mononucleosis-like syndromes were ruled out with Epstein-Barr virus (EBV) viral capsid antigen, and monospot IgM was negative, cytomegalovirus (CMV) IgM was positive with titers 14.76 NTU (normal <9 NTU), but quantitative real-time polymerase chain reaction (PCR) for CMV was negative. Peripheral blood smear showed clumped masses of RBC with an increased reticulocyte count of 5.4% (Figures 1, 2), suggesting hemolytic anaemia; this was confirmed by direct Coombs test positivity.

**Figure 3:**
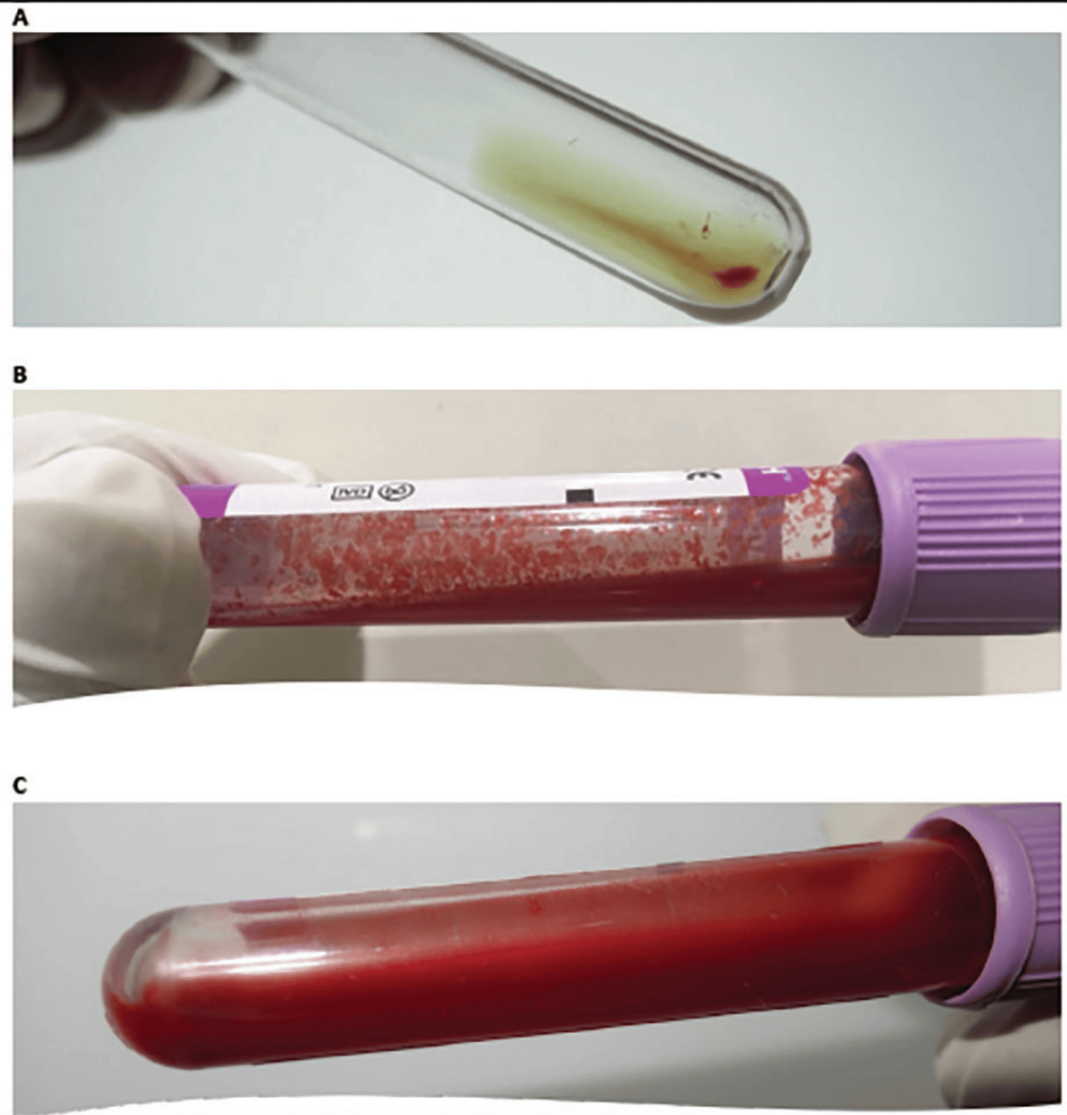
A) Direct Coombs test positive at antibody titre 1:1024. B) At room temperature (18°C), blood specimens in ethylenediamine tetraacetic acid (EDTA) could not be analysed because of the rapid agglutination. C) After incubation at 37°C, blood in EDTA became fluid.

Urine for haemoglobin was negative, suggesting extravascular hemolysis. AIHA type was confirmed with a cold agglutinin test revealing a significant titer of 1.1024 (Figure 3). Antineutrophilic antibodies, rheumatoid factor, and IgM for mycoplasma were negative. Serum protein electrophoresis showed no M-band, ruling out Waldenstrom macroglobulinemia.

Treatment

After it was confirmed that the patient was having the cold type of AIHA, she was started on oral steroids (prednisolone 1 mg/kg with a plan to taper), and a work-up to identify the underlying cause of the disease was done. Her ultrasound neck showed a relatively defined, heterogenous predominant hypoechoic solid, taller than wider lesion of size 4 cm x 4 cm in the right lobe suggestive of a thyroid imaging reporting and data system (TI-RADS) 5 lesion. Another similar lesion was present in the left lobe measuring 0.4 cm x 0.3 cm with peripheral macrocalcifications. USG-guided fine-needle aspiration cytology (FNAC) of the lesions was done, which showed cellular aspirate in a cluster with nuclear crowding and overlapping and moderate to marked atypia in Papanicolaou stain, suggestive of Bethesda class 4.

**Figure 4:**
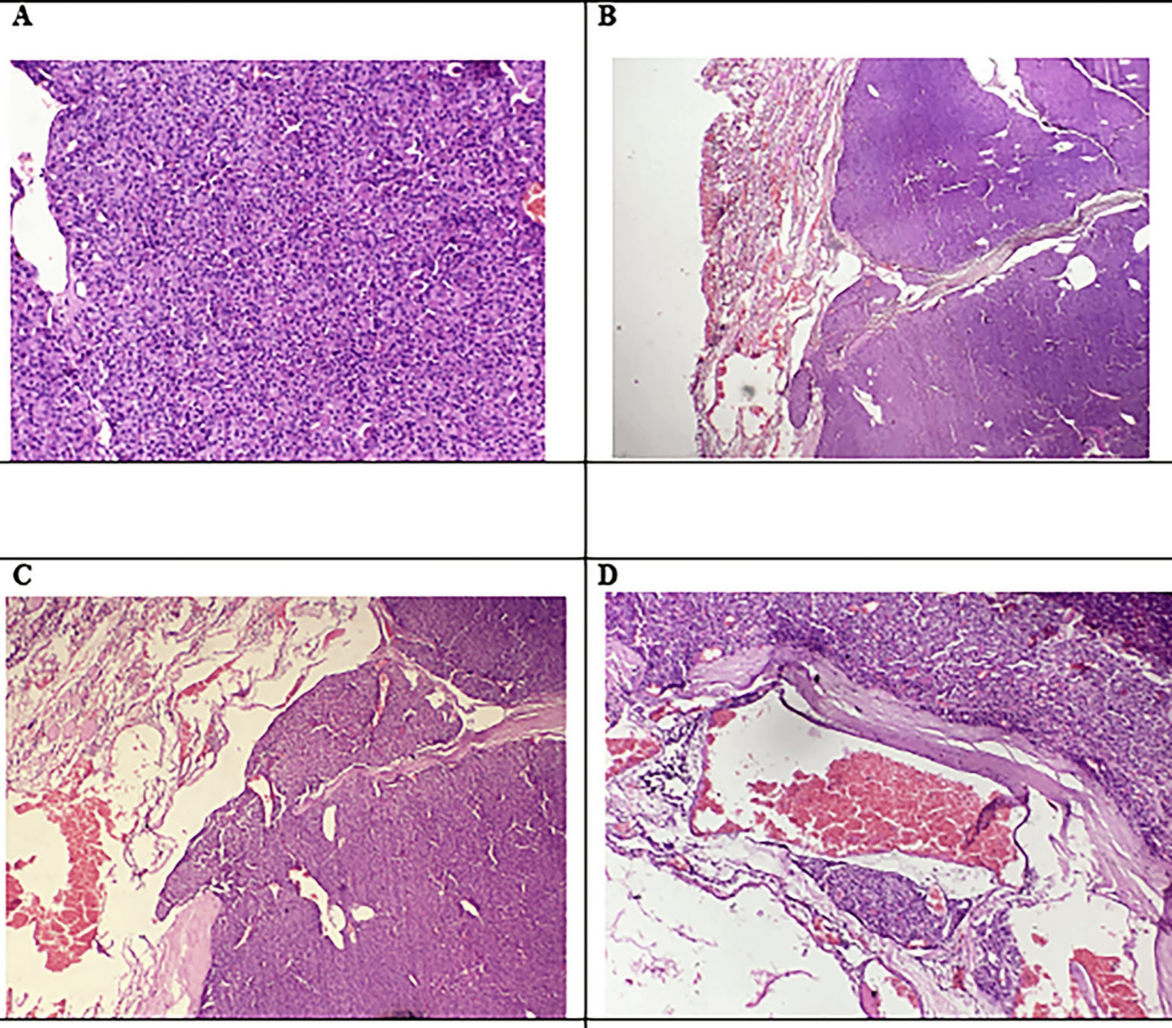
Histopathology of the right thyroid lobe. A) Follicular thyroid carcinoma, oncocytic variant (Hürthle cell carcinoma). Tumour with the growth of solid pattern of follicles (20x, H&E). B) Invasion of adjacent thyroid capsule with complete penetration (4x, H&E). C) Invasion of adjacent thyroid capsule with complete penetration (10x, H&E). D) Vessel beyond capsule with tumour covered with endothelium, attached to the wall (10x, H&E).

**Figure 5:**
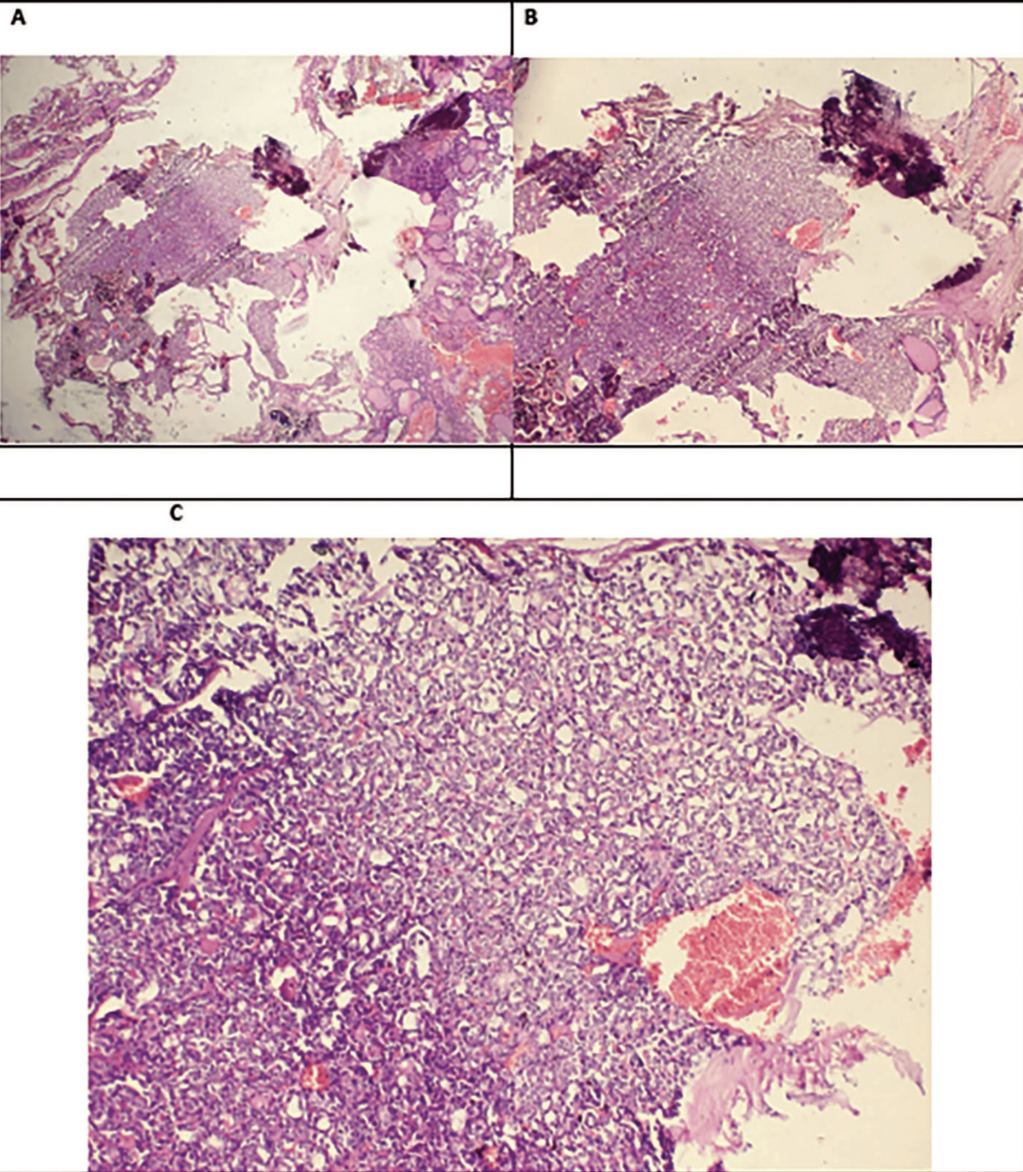
Histopathology of the left lobe of the thyroid. A) Small focus of papillary thyroid carcinoma arranged as microfollicles (2x, H&E). B) Small focus of papillary thyroid carcinoma arranged as microfollicles (4x, H&E). C) Neoplastic cells arranged as microfollicles, some having central colloid. The tumour cell nuclei are enlarged and overlap. They have nuclear grooves, nucleoli, and abundant pseudoinclusions. The chromatin of the tumour cells has a powdery appearance (20x, H&E).

The patient was planned for total thyroidectomy after proper consent and pre-anesthetic check-ups. The gross specimen showed an 8 cm x 6 cm x 5.5 cm thyroid with a solid grey, white growth in the right lobe of size 5.5 cm x 3 cm attached to the capsule; the isthmus was unremarkable, and the left lobe showed a whitish nodular area of 0.5 cm. Histopathology sections revealed follicular thyroid carcinoma (oncolytic variant) (Hürthle cell carcinoma) in the right lobe of the thyroid gland, with capsular and vascular invasion (Figure 4). The left lobe showed micro follicles, with a central colloid with enlarged nuclei with typical nucleolar grooving and abundant pseudo inclusions suggestive of the follicular variant of papillary carcinoma (Figure 5).

Outcome and follow-up

A repeat cold agglutinin test was done, which was negative this time with a negative direct Coombs test. Also, the patient's haemoglobin was increased to 11 g/dl with no residual evidence of hemolytic anaemia. The patient was discharged and referred to the Surgical Oncology and Endocrinology department for further follow-up.

## Discussion

AIHA is an acquired hemolytic anaemia. It is divided into cold and warm types, depending on the temperature at which autoantibodies cross-react with red blood cells [1]. The warm type of hemolytic anaemias is usually idiopathic or drug-associated and shows a positive reaction with anti-IgG and a negative reaction with C3d. In SLE, the warm type of hemolytic anaemia shows a positive reaction with both IgG and C3d, but in CAD, the reaction is negative with anti-IgG and positive with C3d [2]. Also, agglutination occurs at a temperature of around 3°C or in acral parts of the body.

AIHA is a well-documented paraneoplastic syndrome in lymphoproliferative disorders like lymphoma [3]. Rarely, they can occur in solid tumour malignancies like malignancies of the breast, prostate, and thyroid, ovarian dermoid cysts, renal cell carcinoma, and Kaposi sarcoma. A review of the literature we did found 52 such cases mentioned.

The temporal relationship between AIHA and cancers is not well established. It can occur before cancer, concurrent with cancer, only at the relapse of cancer, or after successful treatment during a complete remission period. It can also present both during the early stages and during metastasis [4]. Most cases have been seen concurrent to cancer and in the stage of late cancer.

Thyroid cancer rarely presents with paraneoplastic manifestations like hypercalcemia, polymyalgia rheumatic, paraneoplastic neutrophilia, dermatopolymyositis, neurological symptoms like myoclonus, and optic neuritis [5-12]. One case report mentioned medullary carcinoma thyroid presenting as a hypercoagulable state, marantic endocarditis, and recurrent strokes [13]. Another case report mentions papillary carcinoma of the thyroid with the syndrome of inappropriate antidiuretic hormone secretion (SIADH) as a paraneoplastic phenomenon, which gradually resolved successfully after tumour resection [14].

The response of paraneoplastic syndromes to treatment is variable. Early-stage tumour, if resected, shows complete remission [15]. These are usually resistant to steroids. While another subset of tumours, usually in the late stage, shows a good response to steroids. A patient with recurrent AIHA responds well to chemotherapy. In our case, anaemia improved with steroids followed by tumour resection. Thus, a search for malignancy should be initiated if a diagnosis of AIHA is made.

## Conclusions

CAD is usually associated with lymphoproliferative disorders but rarely with solid tumour malignancies, such as thyroid cancer. CAD can be a paraneoplastic manifestation of an underlying malignancy. The follow-up of these patients is necessary, as the disease may recur with the recurrence of malignancy. The association between CAD and solid tumours underscores the importance of comprehensive cancer screening in affected patients. Clinicians should remain vigilant for signs of malignancy, particularly in cases where CAD appears to be idiopathic. Long-term monitoring of patients with CAD is crucial, as it may serve as an early indicator of cancer recurrence or progression.
